# Plasma Lipidomic Profiling of Treated HIV-Positive Individuals and the Implications for Cardiovascular Risk Prediction

**DOI:** 10.1371/journal.pone.0094810

**Published:** 2014-04-14

**Authors:** Gerard Wong, Janine M. Trevillyan, Benoit Fatou, Michelle Cinel, Jacquelyn M. Weir, Jennifer F. Hoy, Peter J. Meikle

**Affiliations:** 1 Baker IDI Heart and Diabetes Institute, Melbourne, Victoria, Australia; 2 Infectious Diseases Unit, Alfred Hospital, Melbourne, Victoria, Australia; 3 Department of Infectious Diseases, Faculty of Medicine, Nursing and Health Science, Monash University, Victoria, Australia; 4 University of Sciences and Technologies of Lille, Lille France; Instituto de Investigación Sanitaria INCLIVA, Spain

## Abstract

**Background:**

The increased risk of coronary artery disease in human immunodeficiency virus (HIV) positive patients is collectively contributed to by the human immunodeficiency virus and antiretroviral-associated dyslipidaemia. In this study, we investigate the characterisation of the plasma lipid profiles of treated HIV patients and the relationship of 316 plasma lipid species across multiple lipid classes with the risk of future cardiovascular events in HIV- positive patients.

**Methods:**

In a retrospective case-control study, we analysed plasma lipid profiles of 113 subjects. Cases (n = 23) were HIV-positive individuals with a stored blood sample available 12 months prior to their diagnosis of coronary artery disease (CAD). They were age and sex matched to HIV-positive individuals without a diagnosis of CAD (n = 45) and with healthy HIV-negative volunteers (n = 45).

**Results:**

Association of plasma lipid species and classes with HIV infection and cardiovascular risk in HIV were determined. In multiple logistic regression, we identified 83 lipids species and 7 lipid classes significantly associated with HIV infection and a further identified 74 lipid species and 8 lipid classes significantly associated with future cardiovascular events in HIV-positive subjects. Risk prediction models incorporating lipid species attained an area under the receiver operator characteristic curve (AUC) of 0.78 (0.775, 0.785)) and outperformed all other tested markers and risk scores in the identification of HIV-positive subjects with increased risk of cardiovascular events.

**Conclusions:**

Our results demonstrate that HIV-positive patients have significant differences in their plasma lipid profiles compared with healthy HIV-negative controls and that numerous lipid species were significantly associated with elevated cardiovascular risk. This suggests a potential novel application for plasma lipids in cardiovascular risk screening of HIV-positive patients.

## Introduction

With advances in antiretroviral therapy (ART) and the management of HIV disease there have been substantial improvements in disease-free survival for individuals with HIV [1]. These improvements have resulted in a rapidly growing population of older patients living with HIV. As a result, the major burden of illness, health care utilization and premature death in HIV-positive patients is now due to diseases of ageing, particularly cardiovascular disease (CVD), which occurs not only at higher rates (2-fold increased relative risk) but also at a younger age than in the general population [2], [3]. This elevated cardiovascular risk is due to a complex interplay between the increased prevalence of traditional cardiovascular risk factors [4], ART-related toxicity [5] and the pro-inflammatory actions of HIV itself [6].

Disequilibrium in lipid metabolism is key to the formation and rupture of the atherosclerotic plaques which underpin coronary artery disease (CAD). ART-induced dyslipidaemia is classically characterised by elevated triglycerides, total cholesterol and low-density lipoprotein cholesterol (LDL-C), with reduced high-density lipoprotein cholesterol (HDL-C) [7], a distinctly atherogenic lipid pattern. Yet these standard measures of cholesterol and triglycerides do not fully reflect the complex changes in lipid metabolism induced by HIV and ART. Thus risk equations (such as the Framingham or Reynolds risk scores) which do not account for these complex changes or the effect of HIV and ART induced immune activation on CVD risk are likely to underestimate the risk in this population [8], [9].

Attempts to rectify this by incorporating HIV factors such as the duration of exposure to specific antiretrovirals into traditional risk scores have to date not contributed significantly to the overall accuracy of risk predication models [10]. Given the ageing HIV population and increased rates of cardiovascular disease, improved CAD prediction in HIV patients is urgently needed to guide intensive risk factor modification and ARV choice in those at highest risk. Plasma lipid profiling, where several hundred lipid species are measured using mass spectrometry has the potential to define the underlying changes in lipid metabolism and has shown promise for the enhanced prediction of patients at risk for coronary events [11], but has not previously been explored in the context of HIV.

This study aims to describe the differences in plasma lipid profiles between HIV-positive patients with and without CAD and healthy participants so as to develop and cross-validate a predictive model for future cardiovascular events in HIV-positive patients.

## Methods

### Ethics Statement

Participants provided written informed consent for the original study and for their samples to be stored and used for future research. This project received ethics approval from the Alfred Hospital Research and Ethics Committee (Project number 205/09).

### Participants

This study was based on a subset of patients drawn from a retrospective case- control study on HIV-positive patients who were seen at the Alfred Hospital, Melbourne, Australia from January 1996 to December 2009. The aim of original study was to determine risk factors for the development of CAD in the Australian context. A detailed description of the methods and results have been described elsewhere [12]. In brief, cases were HIV-positive with a first diagnosis of acute myocardial infarction (AMI), positive coronary angiogram or clinical diagnosis of CAD (angina with consistent electrocardiogram). These diagnoses are collectively referred to as cardiovascular events. Two HIV-positive controls were assigned for each case. The controls, selected from the Alfred Hospital HIV database, were age (within 2 years) and sex matched to the cases and had no history of CAD. Standard demographics, documented cardiovascular risk factors (family history of CAD, diabetes, smoking status, hypertension), use of risk-modifying medication prior to CAD diagnosis and HIV characteristics (duration of known infection, CD4+ T-cell count, HIV viral load (VL) and antiretroviral exposure) were collected from the HIV database and from a manual review of medical records.

Stored specimens were retrieved from The Victorian HIV Blood and Tissue Storage Bank (VHBTSB) (prospective collection of over 25,000 samples from more than 1100 HIV-positive patients with written consent since 1996). From the original study, 23 HIV cases had stored plasma samples available. The 23 cases were frequency-matched for age, sex, body mass index (BMI) and systolic blood pressure to 45 HIV controls for lipidomic profiling. Samples from 45 HIV-negative healthy subjects with no prior history of cardiovascular disease (healthy controls) were drawn from the Baker IDI Heart and Diabetes Institute biobank and also frequency- matched to the HIV controls for age, sex, BMI, systolic blood pressure and date of sample collection.

Lipidomic analysis of 316 lipid species from 24 lipid classes was performed as previously described [13] on 113 baseline samples (23 HIV cases, 45 HIV controls and 45 healthy controls), where the baseline is approximately one year (median (IQR) of 1.01 (0.96-1.08)) prior to the first diagnosis of CAD in HIV cases or equivalently of a cardiovascular event as described earlier. Details of the experimental protocol are described (File S1).

### Statistical analyses

Multiple binary logistic regression was applied to examine the association of plasma lipid species with HIV infection independent of cardiovascular risk. This analysis was based on the HIV control group and the healthy control group adjusting for significant non-lipid related characteristics (family history of CVD, smoking and hsCRP) between the groups. To examine the association of plasma lipid species with cardiovascular risk in HIV subjects we applied multiple binary logistic regression adjusting for significant non-lipid related characteristics (current statin treatment) between the HIV case and HIV control groups. The odds ratios obtained in these analyses represent a change in the risk of being associated with an outcome (either being HIV-positive or having a future cardiovascular event if HIV-positive) corresponding to an interquartile range increase in lipid species measurement. The p-values were corrected for multiple comparisons using the Benjamini-Hochberg method [14]. A corrected p-value of <0.05 was considered to be statistically significant.

Models were developed to predict future cardiovascular events in HIV-positive subjects through the application of statistical machine learning approaches similar to that applied in previous related work [11], [15], [16]. The prediction task was the discrimination of HIV cases from HIV controls. The feature sets used in model development were based on lipid species alone, conventional lipids alone (HDL-C, LDL-C, total cholesterol and triglycerides) and a combination of lipid species and conventional lipids. Modeling was performed within a 3-fold class-stratified cross-validation framework utilizing univariate area under the ROC curve (AUC) feature selection for prioritising features to be included into a support vector machine-based classification model [17]. Model development was performed in MATLAB 2012b. For 3-fold cross-validation, the dataset was split into thirds. The model was trained using two thirds of the data and the performance of the trained model was tested on the remaining third of samples. The same proportions of HIV cases and HIV controls were maintained in each third. The thirds of samples were permutated three times in each iteration and thus in each instance a different two thirds of the dataset were used for training and a separate third for testing the samples. When this was completed, the samples were randomly reallocated into thirds and training and testing were repeated. This process was repeated 400 times and thus 1200 trials were performed. In each trial, we recorded the following measures of performance: percentage accuracy, area under the ROC curve (AUC), sensitivity, specificity, positive predictive value and negative predictive value. We also computed the means and corresponding 95% confidence intervals for these measures. Independently, we calculated the area under the receiver operator characteristic curve (C-statistic) achieved with the Framingham 10-year coronary heart disease (CHD) risk equation [8], the Reynolds 10-year CHD risk equation [9] and the Data Collection on Adverse Events of Anti-HIV Drugs (D:A:D) HIV specific 10-year CHD risk equation [10] respectively in predicting HIV patients who did experience a cardiovascular event over the course of this study from those who did not.

Participant characteristics for each group are described in Table 1. Over 90% were male, with a median age of 51 years for the HIV cases and HIV controls and 54 years for healthy controls. This is a reflection of the HIV cohort managed at The Alfred Hospital. There was a significantly greater proportion of smokers in the HIV groups (52% HIV cases and 42% HIV controls) compared with the healthy controls in whom only 6.7% smoked. Diabetes occurred more frequently in the HIV cases (17.4%) compared with 6.7% in the HIV controls although this was not statistically significant and was 0% in healthy controls. HDL and triglycerides were statistically significant between the HIV groups and healthy controls, with lower HDL levels and higher triglyceride levels observed in the HIV groups. hsCRP was also significantly higher in the HIV groups.

**Table 1:** Participant characteristics.

Characteristics	HIV Cases (A)^1^	HIV Controls (B)^1^	Healthy Controls (C)^1^	A vs B^2^	B vs C^2^	A vs C^2^
	(n = 23)	(n = 45)	(n = 45)			
Male (%)	91.3 (21/23)	91.1 (41/45)	93.3 (42/45)	1	1	1
Systolic Blood Pressure (mmHg)	120 (120–140)	120 (110–130)	121 (112.25–132.5)	0.13	0.61	0.69
BMI (kg/m^2^)	22.6 (21.1–27.2)	24.3 (21.8–27.7)	23.6 (22.0–26.5)	0.70	0.97	0.86
Family history of CHD (%)	52.2 (12/23)	20.0 (9/45)	0 (0/45)	0.07	**0.004**	**<0.001**
Age (at sample collection)	51.1 (41.0–61.5)	51.2 (40.7–59.1)	54.0 (48.0–57.3)	0.92	0.82	1
Current Smoker (%)	52.2 (12/23)	42.2 (19/45)	6.7 (3/45)	0.66	**0.002**	**0.001**
Type 2 Diabetes (%)	17.4 (4/23)	6.7 (3/45)	0 (0/45)	0.24	0.24	**0.02**
HDL Cholesterol (mmol/L)	0.74 (0.65–1.06)	0.95 (0.78–1.13)	1.50 (1.1–1.7)	0.20	**<0.001**	**<0.001**
LDL Cholesterol (mmol/L)	3.60 (2.83–4.13)	3.30 (2.4–3.7)	2.97 (2.67–3.7)	0.39	0.97	0.17
Triglycerides (mmol/L)	3.67 (2.50–4.56)	1.77 (1.47–2.76)	1.00 (0.7–1.76)	**0.003**	**<0.001**	**<0.001**
Total Cholesterol (mmol/L)	5.89 (5.14–6.79)	5.20 (4.32–5.89)	5.00 (4.55–5.7)	**0.02**	0.99	**0.01**
hsCRP (mg/L)	2.78 (1.97–6.67)	2.26 (1.15–4.23)	0.64 (0.36–1.48)	0.39	**<0.001**	**<0.001**
Current Statin Treatment (%)	34.8 (8/23)	6.7 (3/45)	0 (0/45)	**0.02**	0.24	**<0.001**
Framingham (CHD 10 Year)	17 (11.3–31.5)	9 (5.8–17.0)	6 (4.0–9.2)	**0.02**	**0.03**	**<0.001**
Reynolds (CHD 10 Year)	12 (4.0–20.8)	5 (2.0–11.3)	3 (1.8–5.0)	**0.03**	0.17	**<0.001**
Detectable Viral Load (% ≥50 copies)	39.1 (9/23)	42.2 (19/45)	-	1	-	-
CD4 Count (cells/µL)	452 (310–700)	320 (174–486)	-	0.08	-	-
Currently on ART (%)	100 (23/23)	88.9 (40/45)	-	0.16	-	-
D:A:D (CHD 10 Year)	15.7 (8.1–22.5)	9.4 (3.8–18.8)	-	**0.04**	-	-

1 *median (interquartile range) or percentage (proportion) unless specified otherwise.*

2 *p-values based on Mann-Whitney U tests for continuous variables and Fisher's exact tests otherwise, p-values less than 0.05 are in bold.*

## Results

### Association of plasma lipids with HIV infection

There were six lipid classes (monohexosylceramide, dihexosylceramide, GM3 ganglioside, sphingomyelin, phosphatidylcholine, alkenylphosphatidylcholine) that were negatively associated with HIV while diacylglycerol was positively associated (Table 2). Not all species within a class showed the same association and there were 83 individual lipid species that were significantly associated with HIV infection (Table S1 in File S1). These included 15 individual species of triacylglycerol positively associated with HIV, although the association of the triacylglycerol class was not significant after correcting for multiple comparisons (p = 0.07, Table 2)

**Table 2:** Association of lipid classes with HIV infection and with future cardiovascular events in HIV-positive individuals.

Lipid Class Predictors	Odds Ratio^1^ (HIV infection)	p-value^3^	Odds Ratio^2^ (future cardiovascular events in HIV)	p-value^3^
Dihydroceramide (Cer)	1.36 (0.83–2.22)	0.347	1.61 (1.01–2.58)	0.087
Ceramide (Cer)	0.74 (0.39–1.37)	0.424	**3.44 (1.55**–**7.63)**	0.020
Monohexosylceramide (HexCer)	**0.17 (0.07**–**0.43)**	**0.002**	0.95 (0.44–2.07)	0.902
Dihexosylceramide (Hex2Cer)	**0.31 (0.13**–**0.71)**	**0.026**	1.64 (0.74–3.62)	0.304
Trihexosylcermide (Hex3Cer)	0.54 (0.26–1.14)	0.243	1.25 (0.86–1.83)	0.304
GM3 ganglioside (GM3)	**0.3 (0.14**–**0.62)**	**0.008**	1.17 (0.61–2.25)	0.732
Sphingomyelin (SM)	**0.14 (0.05**–**0.39)**	**0.002**	2.4 (1.02–5.62)	0.087
Phosphatidylcholine (PC)	**0.43 (0.23**–**0.8)**	**0.034**	2.3 (1.14–4.63)	0.061
Alkylphosphatidylcholine (PC O)	0.73 (0.39–1.36)	0.424	0.94 (0.45–1.96)	0.902
Alkenylphosphatidylcholine (plasmalogen) (PC P)	**0.12 (0.04**–**0.33)**	**0.002**	1.46 (0.65–3.27)	0.431
Lysophosphatidylcholine (LPC)	1.23 (0.6–2.51)	0.662	2.74 (1.11–6.77)	0.071
Lysoalkylphosphatidylcholine (LPC O)	2.21 (1.1–4.47)	0.078	1.87 (0.82–4.3)	0.203
Phosphatidylethanolamine (PE)	2.12 (1.04–4.32)	0.098	**4.42 (1.79**–**10.92)**	0.020
Alkylphosphatidylethanolamine (PE O)	0.65 (0.36–1.18)	0.323	2.24 (1.05–4.78)	0.081
Alkenylphosphatidylethanolamine (PE P)	0.88 (0.53–1.45)	0.662	2.03 (1.1–3.77)	0.067
Lysophosphatidylethanolamine (LPE)	0.74 (0.44–1.24)	0.358	**3.07 (1.33**–**7.06)**	0.036
Phosphatidylinositol (PI)	0.71 (0.42–1.21)	0.347	**3.03 (1.53**–**6.03)**	0.020
Lysophosphatidylinositol (LPI)	1.02 (0.63–1.67)	0.926	1.69 (1–2.86)	0.087
Phosphatidylserine (PS)	1.23 (0.9–1.67)	0.347	1.44 (0.94–2.21)	0.144
Phosphatidylglycerol (PG)	1.15 (0.61–2.17)	0.690	**2.73 (1.25**–**5.96)**	0.043
Cholesteryl ester (CE)	1.35 (0.7–2.62)	0.453	**3.62 (1.51**–**8.69)**	0.022
Free cholesterol (ST 27:1/OH)	0.7 (0.39–1.24)	0.347	1 (0.99–1.01)	0.902
Diacylglycerol (DG)	**5.42 (2.06**–**14.22)**	**0.006**	**3.72 (1.55**–**8.9)**	0.022
Triaclyglycerol (TG)	2.48 (1.15–5.38)	0.070	**5.94 (1.89**–**18.66)**	0.020

1 *mean (95% confidence interval) adjusted for family history of CVD, smoking and hsCRP, corresponding to an interquartile range increase in the predictor lipid species measuremen.t*

2 *mean (95% confidence interval) adjusted for current statin treatment, corresponding to an interquartile range increase in the predictor lipid species measurement.*

3 *false discovery rate-corrected p-values using the Benjamini-Hochberg method, p-values less than 0.05 are in bold.*

### Association of plasma lipids with future cardiovascular events in HIV infection

Within the HIV groups, diacylglycerol showed a further positive association with cardiovascular events and this was accompanied by positive associations in seven other lipid classes (ceramide, phosphatidylethanolamine, lysophosphatidylethanolamine, phosphatidylinositol, phosphatidylglycerol, cholesteryl ester and triaclyglycerol) (Table 2). There were 74 individual lipid species that were associated with future cardiovascular events (Table S1 in File S1).

### Prediction of future cardiovascular events in HIV

The performance of all predictive models for future cardiovascular events is presented in Table 3. The Framingham CHD 10-Year Risk, the Reynolds 10-Year Risk of CHD and the D:A:D 10 Year Estimated Risk for CHD achieved an AUC of 0.707, 0.688 and 0.686 respectively. Conventional lipids including HDL, LDL, triglycerides and total cholesterol achieved an AUC of 0.765 and a percentage accuracy of 74.5%. The prediction model based on lipid species performed best with an AUC of 0.78 and a percentage accuracy of 79%. The difference in all measures of performance (percentage accuracy, AUC, sensitivity and specificity) between the lipid species model and the conventional lipids model were statistically significant and in favour of the lipid species model. The lipid species used in this model are shown in Table 4. The combined model with both conventional lipid and lipid species as features (predictors) had an AUC of 0.778 which was not statistically significant in comparison to the lipid species model as the features that were most frequently selected in the top 16 of the combined model consisted only of lipid species and excluded any conventional lipids, implying that lipid species have captured the information available from conventional lipids for the identification of HIV-positive individuals who are at elevated risk of cardiovascular events.

**Table 3:** Summary of predictive model performance for future cardiovascular events in HIV-positive individuals^1^.

Performance Measures	Lipid Species^2^	Conventional Lipids^3^	Combined Lipids^4^	Framingham (CHD 10 Year)^5^	Reynolds (CHD 10 Year)^5^	D:A:D (CHD 10 Year)^5^
No. of features in model^6^	18	4	16	-	-	-
Accuracy	79 (78.6, 79.4)	74.5 (74.1, 74.8)	78.3 (77.9, 78.7)	-	-	-
Area Under ROC Curve	0.78 (0.775, 0.785)	0.765 (0.76, 0.77)	0.778 (0.773, 0.783)	0.707	0.688	0.686
Sensitivity	57.5 (56.7, 58.4)	51.6 (50.7, 52.5)	56.1 (55.2, 57)	-	-	-
Specificity	90 (89.6, 90.4)	86.2 (85.8, 86.6)	89.7 (89.3, 90.1)	-	-	-
Positive Predictive Value	76.3 (75.5, 77.2)	66.8 (66, 67.7)	74.9 (74, 75.8)	-	-	-
Negative Predictive Value	80.9 (80.6, 81.2)	78.1 (77.8, 78.4)	80.4 (80, 80.7)	-	-	-

1 *value (95% confidence intervals).*

2 *268 lipid species distributed across 24 lipid classes.*

3 *HDL-C, LDL-C, total cholesterol and triglycerides.*

4 *conventional lipids and lipid species as features in the mode.*

5 *non cross-validated results.*

6 *number of features required to maximise the mean AUC of the model.*

**Table 4:** Cross-validation feature inclusion frequency (lipid species model).

Rank	Lipid Species^1^	Inclusion Frequency (%)^2^
1	TG 16:1_17:0_18:1	95
2	TG 16:1_18:1_18:1	93.7
3	TG 16:0_17:0_18:2	88.8
4	TG 14:1_18:0_18:2	88.5
5	TG 16:0_18:1_18:1	82
6	TG 17:0_18:1_18:1	81.7
7	TG 16:1_16:1_18:1	78.5
8	TG 16:0_16:1_18:1	71.7
9	CE 16:2	62
10	TG 15:0_18:1_18:1	61.2
11	DG 16:1_18:1	59.7
12	TG 16:1_16:1_16:1	56.3
13	CE 20:2	49.7
14	Cer d18:1/20:0	45.8
15	TG 14:1_18:1_18:1	43.8
16	PE 38:3	32.5
17	TG 16:0_17:0_18:1	31.8
18	PC 36:0	30

1 *TG, triacylglycerol; CE cholesteryl ester; DG, diacylglycerol; Cer, ceramide; PE phosphatidylethanolamine; PC, phosphatidylcholine.*

2 *frequency of feature inclusion in cross-validation over 1200 iterations (3-fold, 400 repeats).*

## Discussion

Dyslipidemia is well described and common in patients with HIV infection on ART. In our study, we describe the significant differences in plasma lipid profiles between HIV-positive patients and healthy HIV-negative volunteers, whilst also identifying lipid species significantly associated with future cardiovascular events in HIV-positive patients.

Increased de novo lipogenesis, the process by which acetyl-CoA is converted to fatty acids, has previously been observed in HIV [18]. Rasheed et al. [19] subsequently demonstrated that HIV replication enhanced the production of free fatty acids in vivo and identified 18 differentially expressed proteins in HIV-infected cells and six enzymes that were expressed exclusively in HIV-infected cells. All of the identified enzymes were known to be essential for the fatty acid synthesis as well as for numerous molecular interactions necessary for lipid metabolism. In this study, we observed a significant positive association of diacylglycerol (odds ratio  = 3.72 (1.55-8.9), p = 0.022) with HIV infection which may be the result of increased circulating concentrations of free (non-esterified) fatty acids resulting in the elevated de novo synthesis of diacylglycerol.

**Figure 1:**
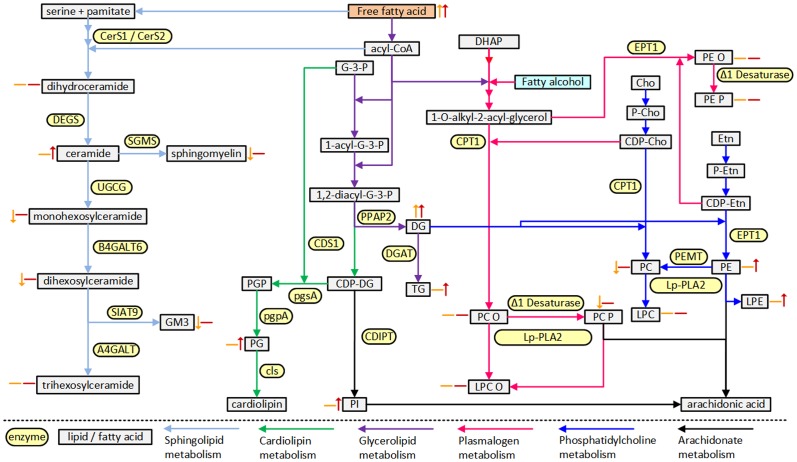
Association of lipid classes with HIV infection and with future cardiovascular events in HIV infection mapped onto key metabolic pathways. Lipid classes are denoted by beige rectangles, enzymes are denoted by yellow rectangles with rounded edges, coloured arrows denote the various lipid metabolic pathways. The association with HIV infection is denoted by orange arrows and the association with future cardiovascular events in HIV is denoted by red arrows. Metabolite abbreviations: Cho, choline; DG, diacylglycerol; DHAP, dihydroxyacetonephosphate; Etn, ethanolamine; LPC, lysophosphatidylcholine; LPC O, lysoalkylphosphatidylcholine; LPE, lysophosphatidylethanolamine; PC, phosphatidylcholine; PC O, alkylphosphatidylcholine; PC P, alkenylphosphatidylcholine; PE, phosphatidylethanolamine; PE O, alkylphosphatidylethanolamine; PE P, alkenylphosphatidylethanolamine; PG, phosphatidylglycerol; PGP, phosphatidylglycerolphosphate; PI, phosphatidylinositol; TG, triacylglycerol. Enzyme abbreviations: A4GALT, lactosylceramide 4-alpha-galactosyltransferase; B4GALT6, beta-1,4-galactosyltransferase 6; CDIPT, CDP-diacylglycerol-inositol 3-phosphatidyltransferase; CDS1, phosphatidate cytidylyltransferase; CerS, ceramide synthasecls, cardiolipin synthase; CPT1, diacylglycerol cholinephosphotransferase; DEGS, sphingolipid delta-4 desaturase; DGAT, diacylglycerol O-acyltransferase; EPT1, diacylglycerol ethanolaminephosphotransferase; PEMT, phosphatidylethanolamine N-methyltransferase; ethanolaminephosphotransferase, pgpA, phosphatidylglycerophosphatase A; pgsA, CDP-diacylglycerol—glycerol-3-phosphate 3-phosphatidyltransferase; PPAP2, phosphatidate phosphatase; SMGS, sphingomyelin synthase; UGCG, ceramide glucosyltransferase.

Interestingly, several lipid classes were found to be negatively associated with HIV infection, these included monohexosylceramide, dihexosylceramide, GM3 ganglioside and sphingomyelin all of which are metabolites of ceramide and suggest a possible dysregulation of sphingolipid metabolism in HIV infection (Figure 1), albeit ceramide itself was not significantly associated with HIV infection. The observed negative association of alkenylphosphatidylcholine (plasmalogen) with HIV infection may relate to an increase in oxidative stress associated with HIV[20] as plasmalogens are reported to be susceptible to oxidation by free radicals associated with oxidative stress. We did not observe a similar negative association with alkenylphosphatidylethanolamine (plasmalogen) and this may reflect the upregulation of the phosphatidylethanolamine pathway observed (odds ratio 2.12, non-significant) also influencing the alkenylphosphatidylethanolamine and so counteracting the effect of oxidative stress.

Oxidative stress in HIV disease has previously been characterised in Pace et al. [21] by elevated serum levels of hydroperoxides and malondialdehyde even in asymptomatic HIV-infected patients in the early stages of the disease. Oxidative stress is thought to contribute to the HIV disease pathogenesis by affecting viral replication, inflammatory response, loss of immune function and increasing sensitivity to drug toxicities. Plasmalogens are also found primarily in HDL particles and so the lower HDL cholesterol in the HIV groups may also be contributing to this observed association [22], [23].

The observed positive association of diacylglycerol with both HIV infection and future cardiovascular events and triacylglycerol with future cardiovascular events appear to be consistent with previous findings [24] which report an early reduction in HDL-cholesterol followed by a subsequent increase in triglycerides and very low density lipoprotein (VLDL) cholesterol in the later stage of HIV infection. Hypertriglyceridemia in HIV is contributed to via a combination of hepatic VLDL overproduction and reduced triglyceride clearance. This is related to poor virological control and increased levels of TNF-α which interferes with free fatty acid metabolism and lipid oxidation and attenuates insulin-mediated suppression of lipolysis [25].

The alterations of fatty acid metabolism leading to elevated free fatty acid and subsequently elevated diacylglycerol and triacylglycerol appear to be exacerbated in individuals who progress to the development of CAD and subsequent cardiovascular events. This also appears to affect other lipid metabolic pathways; while we observed a decrease in several ceramide metabolites associated with HIV infection, in those individuals who subsequently experienced a cardiovascular event we observe a positive association with ceramide itself that may be driven by a dual effect of increased free fatty acid leading to an increased ceramide production and a down regulation of ceramide conversion into downstream metabolites. Further effect of free fatty acids driving the production of phosphatidylethanolamine, phosphatidylglycerol and phosphatidylinositol were also observed (Figure 1). We have recently reported similar associations in type 2 diabetes where multiple metabolic pathways were dysregulated under the influence of elevated free fatty acids [26].

Antiretroviral (ARV) therapy including the use of protease inhibitors (PIs), nucleoside reverse transcriptase inhibitors (NRTIs), non-nucleoside reverse transcriptase inhibitors (NNRTIs) and fusion inhibitors are known to have different effects on the plasma lipid profile [27], [28], [29]. PI therapy has been shown to be associated with an increase in hepatic triglyceride synthesis through a mediated increase in expression of key enzymes involved in the biosynthesis of triglycerides [30]. PIs have also been associated with raising VLDL cholesterol resulting from a reduction in the catabolism of VLDL [31] and an increase in VLDL production [32], [33].

We postulate that a patient's lipidomic response to HIV infection and HIV therapy can vary based on a number of currently unidentified factors and that patients who develop a more significant elevation of diacylglycerols and dysregulation of other related metabolic pathways are at higher risk of future cardiovascular events.

Risk prediction models based on existing cardiovascular risk scores demonstrated limited performance in predicting cardiovascular events in the HIV-positive participants included in this study. The D:A:D (CHD 10 year) risk score which takes into account exposure to specific antiretroviral therapies in addition to traditional CAD risk factors, the Framingham (CHD 10 year) score and the Reynolds (CHD 10 year) scores were comparable in performance (AUC of 0.686, 0.707 and 0.688 respectively) and did not perform as well as predictive models based on conventional lipid predictors (HDL-C, LDL-C, triglycerides and total cholesterol). The conventional lipid model performed well (AUC  = 0.765 (0.76, 0.77) and an accuracy  = 74.5% (74.1%, 74.8%)) in reflecting the more severe dyslipidemia in HIV-positive subjects who experienced future cardiovascular events in this study. However, the lipid species model performed best with an AUC of 0.78 (0.775, 0.785) and an accuracy of 79% (78.6%, 79.4%) as it exploits more specific markers of the dyslipidemia at the individual species level.

In conclusion, HIV-positive patients demonstrate significant abnormalities in their plasma lipid profiles compared with healthy volunteers. The degree of these changes appears to relate to the risk of future cardiovascular events. Predictive models of future cardiovascular events based on plasma lipid profiles demonstrate the potential to outperform traditional risk equations in HIV populations which, if confirmed in larger trials, may allow for the accurate, targeted utilisation of primary preventative strategies. The strongest markers associated with elevated cardiovascular risk in HIV-positive patients were identified to be diacylglycerol and triacylglycerol species where individual species outperform the clinical measure of triglycerides. This suggests the possible dysregulation of the triacylglycerol biosynthetic pathway and/or VLDL metabolism where individuals at greater risk display a more severe elevation in particular triacylglycerol species. This observation may have therapeutic implications and warrants further investigation into the effect of treatment of hypertriglyceridemia in HIV-positive subjects on diacylgylcerol and triacylglycerol levels. An extended study looking at the impact of individual antiretroviral regimens on plasma lipidome may further advance our understanding of the pathogenesis of ART-associated cardiovascular risk and allow for individualised treatment decisions in those at high risk.

### Study Limitations

Statistical power is limited in this study due to its small sample size. Most HIV-positive participants in this study were treatment-experienced, but as these proportions were matched between the HIV cases and controls and we cannot comment on the individual contributions of HIV infection and ART to changes in lipid species. The study group were mostly male (91%) and so further studies are required to validate these findings in females. While the associations identified in the analysis are expected to hold in larger population-based studies, the predictive model for future cardiovascular events is proof-of-concept and does not represent a final model for clinical utility. The model does however demonstrate the potential gain in predictive performance that may be achieved through the use of plasma lipid species as novel biomarkers of cardiovascular risk over the use of conventional clinical lipids and existing cardiovascular risk scores.

## Supporting Information

File S1 **This supplementary material contains Protocol S1 and Table S1.** Experimental Protocol. Sample preparation and lipid extraction. High performance liquid chromatography-mass spectrometry analysis. Assay performance. **Table S1.** Association of lipid species and lipid classes with future cardiovascular events in HIV positive individuals and HIV infection.
(DOCX)
