# Supplementary material for: Plasma Lipidomic Profiling of Treated HIV-Positive Individuals and the Implications for Cardiovascular Risk Prediction
Source: PLoS One. 2014 Apr 14;9(4):e94810. doi: 10.1371/journal.pone.0094810 (PMC3986244; doi:10.1371/journal.pone.0094810)
Supplement: File S1 — This supplementary material contains Protocol S1 and Table S1. Experimental Protocol. Sample preparation and lipid extraction. High performance liquid chromatography-mass spectrometry analysis. Assay performance. Table S1. Association of lipid species and lipid classes with future cardiovascular events in HIV positive individuals and HIV infection. (DOCX) [file pone.0094810.s001.docx]

**Online Supplemental Material**

**Plasma Lipidomic Profiling of Treated HIV-Positive Individuals and the Implications for Cardiovascular Risk Prediction**

Gerard Wong^1^, Janine M. Trevillyan^2,3^, Benoit Fatou^1,4^, Michelle Cinel^1^, Jacquelyn M Weir^1^, Jennifer F. Hoy^2,3^ and Peter J. Meikle^1^

^1^ Baker IDI Heart and Diabetes Institute, Melbourne Australia. ^2^ Infectious Diseases Unit, Alfred Hospital, Melbourne Australia. ^3^ Department of Infectious Diseases, Faculty of Medicine, Nursing and Health Science, Monash University, Victoria, Australia. ^4^ University of Sciences and Technologies of Lille, Lille France.

**Address for correspondence:** Peter J. Meikle, Baker IDI Heart and Diabetes Institute, 75 Commercial Road, Melbourne, Victoria 3004, Australia. Tel: (613) 8532 1770, Fax: (613) 8532 1100, email: peter.meikle@bakeridi.edu.au

**Secondary address:** Jennifer F. Hoy, Infectious Diseases Unit, The Alfred Hospital, 81 Commercial Road, Melbourne, Victoria 3004, Australia. Tel: (613) 9076 6900, e-mail: jennifer.hoy@monash.edu

**SUPPLEMENTARY MATERIAL**

**Experimental Protocol**

**Sample preparation and lipid extraction.** The plasma samples were randomised for lipid extraction and analysis to minimise experimental bias within the groups. Quality control plasma samples were included at a ratio of 1:10. Total lipid extraction from a 10μL aliquot of plasma was performed by a single phase chloroform:methanol (2:1) extraction[^1^](#_ENREF_1).

Briefly, a 10µL aliquot of plasma was combined with 200 µL CHCl_3_/MeOH (2:1) and 15µL of internal standard mix. The internal standards comprised lipids which are either stable isotope labeled or non-physiological and so present in plasma at extremely low concentrations. The samples were briefly vortexed, mixed (rotary mixer, 10 min), sonicated (water bath, 30 min) then allowed to stand (20 min) at room temperature. Samples were centrifuged (16,000×g, 10 min) and the supernatant was dried under a stream of nitrogen at 40^o^C. The extracted lipids were resuspended in 50µL H_2_O saturated BuOH with sonication (10 min), followed by 50µL of 10 mM NH_4_COOH in MeOH. Extracts were centrifuged (3,350xg, 5 min) and the supernatant transferred into 0.2 mL glass vials with teflon insert caps.

**High performance liquid chromatography-mass spectrometry analysis.** Lipid analysis was performed by liquid chromatography, electrospray ionisation-tandem mass spectrometry using a Agilent 1200 liquid chromatography system combined with an Applied Biosystems API 4000 Q/TRAP mass spectrometer with a turbo-ionspray source (350^o^C) and Analyst 1.5 data system. Liquid chromatography was performed on a Zorbax C18, 1.8 µm, 50 × 2.1 mm column (Agilent Technologies). Solvents A and B consisted of tetrahydrofuran:methanol:water in the ratio (30:20:50) and (75:20:5) respectively, both containing 10 mM ammonium formate. Columns were heated to 50^o^C and the auto-sampler regulated to 25^o^C. Diacylglycerol and triacylglycerol species (1 µL injection) were separated using an isocratic flow (100 µL/min) of 85% B over 6 minutes. All other lipid species (5μL injection) were separated under gradient conditions (300 µL/min) 0% B to 100% B over 8.0 min, 2.5 min at 100% B, a return to 0% B over 0.5 min then 10.5 min at 0% B prior to the next injection.

We have previously reported the use of precursor ion and neutral loss scans on control plasma extracts to identify the predominant lipid species of the following lipid classes: dihydroceramide (Cer), ceramide (Cer), monohexosylceramide (HexCer), dihexosylceramide (Hex2Cer), trihexosylcermide (Hex3Cer), GM3 ganglioside (GM3), sphingomyelin (SM), phosphatidylcholine (PC), alkylphosphatidylcholine (PC O), alkenylphosphatidylcholine (plasmalogen, PC P), lysophosphatidylcholine (LPC), lysoalkylphosphatidylcholine (lysoplatelet activating factor, LPC O), phosphatidylethanolamine (PE), alkylphosphatidylethanolamine (PE O), alkenylphosphatidylethanolamine (plasmalogen, PE P), lysophosphatidylethanolamine (LPE), phosphatidylinositol (PI), lysophosphatidylinositol (LPI), phosphatidylserine (PS), phosphatidylglycerol (PG), cholesteryl ester (CE), free cholesterol (ST 27:1/OH), diacylglycerol (DG) and triaclyglycerol (TG)[^1-4^](#_ENREF_1).

Multiple reaction monitoring (MRM) experiments, established for each lipid species, were combined into two scheduled MRM experiments (sMRM) whereby data from each MRM was only collected during its retention time window (± 30sec). Comparative lipid abundances were calculated by relating the peak area of each species to the peak area of the corresponding internal standard. Peak integration was performed using AB Sciex MultiQuant software v1.2. Total measured lipids of each class were calculated by summing the abundance of individual lipid species.

The assignment of isobaric peaks in particular odd-chain diacyl species as well as alkyl and alkenyl species of phosphatidylcholine and phosphatidylethanolamine were made based on product ion scan in both positive and negative mode (to differentiates isobaric species containing acyl linked odd chain fatty acids from those containing alkyl and alkenyl linkages) and relative retention time compared to a series of alkyl and alkenyl standards (Avanti polar lipids and in-house synthesised standards).

The abbreviations shown above are only used when referring to individual lipid species e.g. LPC 22:6 which defines a lysophosphatidylcholine with a fatty acid containing 22 carbons and six double bonds. For a number of the lipids which contain two fatty acid chains the mass spectrometry based measurements here do not directly determine the constituent fatty acids but rather the sum of the number of carbons and the sum of the number of double bonds across both fatty acids. Accordingly, we denote these species as the combined length and number of double bonds, e.g. PC 36:4.

Multiple Reaction Monitoring (MRM) experiments were established for the major species of each lipid class identified in plasma. Although not the major fragmentation pathway, phosphatidylethanolamine plasmalogens were analysed using MRMs based on the neutral loss of 141 as the standard used for these was PE 17:0/17:0 which does have a neutral loss of 141 as the major fragmentation pathway. We recognise that this will affect accuracy of the phosphatidylethanolamine plasmalogen measurements; however, our goal in this study was to achieve precise relative measures of many lipid species and so we have used a common fragmentation for all phosphatidylethanolamine species that are related to the same internal standard.

A total of 63 diacylglycerol (19) and triacylglycerol (44) species and 253 lipid species distributed across another 24 lipid classes were analysed in two separate experiments. Relative lipid levels were calculated by relating the peak area of each species to the peak area of the corresponding stable isotope or non-physiological internal standard. Total lipid class levels were calculated as the sum of the individual lipid species levels within each class.

**Assay performance.** Based on the analysis of the 9 quality control samples, 82% of lipid species (216) had a percentage coefficient of variation (%CV) of less than 20% and 90% of lipid species (237) had a %CV less than 30%. Lipid species with a %CV of more than 50% (10) were primarily low abundance plasma lipid species with a high signal-to-noise ratio. These species were excluded from our statistical analysis list as their reported measurements were deemed to be unreliable.

**References**

1. Weir JM, Wong G, Barlow CK, Greeve MA, Kowalczyk A, Almasy L, Comuzzie AG, Mahaney MC, Jowett JB, Shaw J, Curran JE, Blangero J, Meikle PJ. Plasma lipid profiling in a large population-based cohort. *Journal of lipid research*. 2013;54:2898-2908

2. Meikle PJ, Wong G, Tsorotes D, Barlow CK, Weir JM, Christopher MJ, MacIntosh GL, Goudey B, Stern L, Kowalczyk A, Haviv I, White AJ, Dart AM, Duffy SJ, Jennings GL, Kingwell BA. Plasma lipidomic analysis of stable and unstable coronary artery disease. *Arteriosclerosis, thrombosis, and vascular biology*. 2011;31:2723-2732

3. Borg ML, Andrews ZB, Duh EJ, Zechner R, Meikle PJ, Watt MJ. Pigment epithelium-derived factor regulates lipid metabolism via adipose triglyceride lipase. *Diabetes*. 2011;60:1458-1466

4. Boslem E, MacIntosh G, Preston AM, Bartley C, Busch AK, Fuller M, Laybutt DR, Meikle PJ, Biden TJ. A lipidomic screen of palmitate-treated min6 beta-cells links sphingolipid metabolites with endoplasmic reticulum (er) stress and impaired protein trafficking. *The Biochemical journal*. 2011;435:267-276

**Table S1. Association of lipid species and lipid classes with future cardiovascular events in HIV positive individuals and HIV infection**

| **Predictor^1^** | **Odds Ratio^2^**  **(HIV infection)** | **p-value^3^** | **Odds Ratio^2^**  **(future cardiovascular events in HIV)** | **p-value^3^** |
| --- | --- | --- | --- | --- |
|  |  |  |  |  |
| Cer d18:0/18:0 | 2.27 (1.17-4.41) | 0.060 | 1.71 (1.06-2.75) | 0.086 |
| Cer d18:0/22:0 | 1.53 (0.98-2.37) | 0.146 | 1.68 (1-2.81) | 0.118 |
| Cer d18:0/24:0 | 1.03 (0.61-1.75) | 0.938 | 1.62 (0.97-2.71) | 0.132 |
| Cer d18:0/24:1 | 1.36 (0.74-2.49) | 0.452 | 1.59 (1-2.53) | 0.119 |
| dihydroceramide | 1.36 (0.83-2.22) | 0.347 | 1.61 (1.01-2.58) | 0.087 |
| Cer d18:1/16:0 | 1.93 (0.98-3.8) | 0.141 | **2.62 (1.34-5.11)** | **0.043** |
| Cer d18:1/18:0 | **7.22 (2.8-18.62)** | **0.004** | 2.4 (1.23-4.7) | 0.052 |
| Cer d18:1/20:0 | **2.65 (1.27-5.51)** | **0.044** | **3.26 (1.4-7.57)** | **0.045** |
| Cer d18:1/22:0 | 1.28 (0.74-2.2) | 0.503 | **2.38 (1.28-4.45)** | **0.045** |
| Cer d18:1/24:0 | 0.55 (0.29-1.04) | 0.162 | 2.67 (1.21-5.89) | 0.066 |
| Cer d18:1/24:1 | 0.71 (0.33-1.53) | 0.515 | **3.5 (1.59-7.71)** | **0.040** |
| ceramide | 0.74 (0.39-1.37) | 0.424 | **3.44 (1.55-7.63)** | **0.020** |
| HexCer d18:1/16:0 | 0.62 (0.32-1.2) | 0.288 | 1.19 (0.56-2.51) | 0.701 |
| HexCer d18:1/18:0 | 0.41 (0.2-0.83) | 0.057 | 0.85 (0.44-1.65) | 0.690 |
| HexCer d18:1/20:0 | **0.38 (0.19-0.75)** | **0.035** | 0.89 (0.44-1.79) | 0.782 |
| HexCer d18:1/22:0 | **0.2 (0.09-0.49)** | **0.008** | 0.9 (0.44-1.84) | 0.801 |
| HexCer d18:1/24:0 | **0.15 (0.06-0.38)** | **0.004** | 0.94 (0.43-2.06) | 0.900 |
| HexCer d18:1/24:1 | **0.16 (0.06-0.44)** | **0.010** | 0.97 (0.43-2.2) | 0.946 |
| monohexosylceramide | **0.17 (0.07-0.43)** | **0.002** | 0.95 (0.44-2.07) | 0.902 |
| Hex2Cer d18:1/16:0 | **0.22 (0.08-0.58)** | **0.019** | 1.36 (0.58-3.19) | 0.563 |
| Hex2Cer d18:1/18:0 | 0.99 (0.54-1.82) | 0.988 | 1.22 (0.66-2.26) | 0.612 |
| Hex2Cer d18:1/20:0 | 1 (0.56-1.79) | 0.997 | 1.73 (1.02-2.94) | 0.106 |
| Hex2Cer d18:1/22:0 | 1.09 (0.64-1.85) | 0.827 | 1.85 (1.08-3.16) | 0.082 |
| Hex2Cer d18:1/24:0 | 0.73 (0.44-1.19) | 0.332 | 1.9 (1.1-3.26) | 0.076 |
| Hex2Cer d18:1/24:1 | 0.39 (0.18-0.85) | 0.068 | 1.19 (0.6-2.39) | 0.682 |
| dihexosylceramide | **0.31 (0.13-0.71)** | **0.026** | 1.64 (0.74-3.62) | 0.304 |
| Hex3Cer d18:1/16:0 | **0.31 (0.13-0.74)** | **0.041** | 1.29 (0.68-2.44) | 0.525 |
| Hex3Cer d18:1/18:0 | 0.47 (0.23-0.98) | 0.117 | 1.24 (0.73-2.1) | 0.505 |
| Hex3Cer d18:1/20:0 | 1.03 (0.67-1.59) | 0.933 | 1.25 (0.86-1.81) | 0.341 |
| Hex3Cer d18:1/22:0 | 1.13 (0.72-1.77) | 0.710 | 1.15 (0.91-1.45) | 0.324 |
| Hex3Cer d18:1/24:0 | 0.73 (0.39-1.36) | 0.452 | 1.22 (0.9-1.67) | 0.292 |
| Hex3Cer d18:1/24:1 | 0.76 (0.42-1.38) | 0.501 | 1.15 (0.84-1.6) | 0.479 |
| trihexosylcermide | 0.54 (0.26-1.14) | 0.243 | 1.25 (0.86-1.83) | 0.304 |
| GM3 d18:1/16:0 | 0.34 (0.14-0.85) | 0.071 | 0.99 (0.42-2.35) | 0.981 |
| GM3 d18:1/18:0 | **0.12 (0.05-0.34)** | **0.004** | 1.14 (0.52-2.52) | 0.779 |
| GM3 d18:1/20:0 | 0.65 (0.37-1.15) | 0.264 | 1.17 (0.64-2.13) | 0.682 |
| GM3 d18:1/22:0 | 0.61 (0.31-1.19) | 0.274 | 1.37 (0.75-2.48) | 0.397 |
| GM3 d18:1/24:0 | **0.25 (0.11-0.57)** | **0.013** | 1.1 (0.64-1.9) | 0.769 |
| GM3 d18:1/24:1 | **0.41 (0.23-0.74)** | **0.024** | 1.09 (0.58-2.05) | 0.813 |
| GM3 ganglioside | **0.3 (0.14-0.62)** | **0.008** | 1.17 (0.61-2.25) | 0.732 |
| SM d31:1 | 0.64 (0.36-1.11) | 0.232 | 1.26 (0.7-2.26) | 0.521 |
| SM d32:1 | 0.64 (0.36-1.13) | 0.245 | 2.18 (1.07-4.45) | 0.094 |
| SM d32:2 | **0.31 (0.14-0.67)** | **0.024** | 1.6 (0.79-3.26) | 0.278 |
| SM d34:1 | **0.32 (0.15-0.71)** | **0.031** | 1.74 (0.84-3.58) | 0.216 |
| SM d34:2 | **0.18 (0.07-0.43)** | **0.005** | 2.06 (0.87-4.87) | 0.175 |
| SM d34:3 | **0.35 (0.17-0.72)** | **0.029** | 1.6 (0.79-3.23) | 0.275 |
| SM d35:1 | 0.74 (0.39-1.4) | 0.490 | 1.36 (0.67-2.72) | 0.487 |
| SM d35:2 | 0.39 (0.18-0.85) | 0.065 | 1.69 (0.79-3.64) | 0.265 |
| SM d36:1 | 0.73 (0.37-1.43) | 0.493 | 1.54 (0.93-2.53) | 0.165 |
| SM d36:2 | **0.31 (0.13-0.69)** | **0.031** | 1.55 (0.8-3) | 0.277 |
| SM d36:3 | **0.22 (0.09-0.55)** | **0.014** | 1.93 (0.88-4.25) | 0.177 |
| SM d37:2 | 0.57 (0.3-1.06) | 0.176 | 1.32 (0.69-2.55) | 0.493 |
| SM d38:1 | **0.17 (0.06-0.47)** | **0.011** | 1.93 (0.82-4.53) | 0.211 |
| SM d38:2 | **0.09 (0.03-0.28)** | **0.004** | **3.11 (1.34-7.22)** | **0.049** |
| SM d39:1 | 0.43 (0.2-0.93) | 0.092 | 1.33 (0.61-2.88) | 0.554 |
| SM d41:1 | **0.16 (0.06-0.47)** | **0.011** | 1.69 (0.82-3.5) | 0.240 |
| SM d41:2 | **0.11 (0.03-0.35)** | **0.007** | 1.44 (0.6-3.47) | 0.505 |
| SM d42:1 | **0.17 (0.07-0.45)** | **0.008** | 2.51 (1.09-5.79) | 0.091 |
| sphingomyelin | **0.14 (0.05-0.39)** | **0.002** | 2.4 (1.02-5.62) | 0.087 |
| PC 28:0 | 0.8 (0.45-1.42) | 0.569 | 1.4 (0.8-2.45) | 0.325 |
| PC 29:0 | 0.87 (0.51-1.48) | 0.715 | 1.62 (0.87-3.01) | 0.207 |
| PC 30:0 | 0.53 (0.26-1.08) | 0.183 | 1.69 (0.97-2.96) | 0.133 |
| PC 31:0 | 0.89 (0.5-1.59) | 0.785 | 2.01 (0.97-4.18) | 0.132 |
| PC 31:1 | 0.55 (0.29-1.03) | 0.153 | 1.58 (0.84-2.94) | 0.233 |
| PC 32:0 | 0.71 (0.39-1.28) | 0.382 | 2.44 (1.12-5.32) | 0.082 |
| PC 33:0 | 0.94 (0.5-1.76) | 0.890 | 2.06 (0.99-4.26) | 0.119 |
| PC 33:1 | 0.78 (0.42-1.47) | 0.569 | **3.72 (1.39-9.98)** | **0.050** |
| PC 33:2 | 0.43 (0.2-0.92) | 0.092 | 2.26 (0.92-5.51) | 0.144 |
| PC 33:3 | **0.25 (0.1-0.63)** | **0.025** | 2.33 (1-5.39) | 0.118 |
| PC 34:0 | **0.3 (0.14-0.63)** | **0.014** | 2.77 (1.27-6.02) | 0.052 |
| PC 34:1 | **0.29 (0.13-0.63)** | **0.018** | 2.45 (1.14-5.27) | 0.077 |
| PC 34:2 | **0.3 (0.14-0.65)** | **0.019** | 1.35 (0.61-3) | 0.539 |
| PC 34:3 | **0.19 (0.08-0.46)** | **0.007** | 2.83 (1.24-6.5) | 0.063 |
| PC 34:4 | 0.4 (0.2-0.83) | 0.055 | 1.8 (0.98-3.33) | 0.131 |
| PC 34:5 | 0.79 (0.53-1.19) | 0.394 | 1.19 (0.76-1.87) | 0.526 |
| PC 35:0 | 1.14 (0.73-1.76) | 0.694 | 2.08 (1.07-4.06) | 0.091 |
| PC 35:3 | 0.6 (0.29-1.24) | 0.298 | **4.47 (1.66-12.04)** | **0.040** |
| PC 35:4 | 1.03 (0.78-1.34) | 0.896 | 0.86 (0.66-1.12) | 0.355 |
| PC 36:0 | 0.96 (0.64-1.45) | 0.891 | **2.89 (1.36-6.13)** | **0.045** |
| PC 36:2 | 0.48 (0.25-0.92) | 0.085 | 1.48 (0.75-2.95) | 0.350 |
| PC 36:3 | 0.71 (0.38-1.32) | 0.407 | **3.73 (1.51-9.19)** | **0.043** |
| PC 36:4 | 0.52 (0.27-0.99) | 0.120 | 1.24 (0.62-2.49) | 0.620 |
| PC 36:5 | 0.73 (0.49-1.09) | 0.245 | 1.15 (0.75-1.75) | 0.608 |
| PC 36:6 | 0.59 (0.34-1.02) | 0.141 | 1.43 (0.89-2.31) | 0.222 |
| PC 37:4 | 1.06 (0.58-1.93) | 0.891 | 1.28 (0.54-3) | 0.650 |
| PC 37:5 | 0.92 (0.63-1.34) | 0.772 | 1.29 (0.81-2.07) | 0.374 |
| PC 37:6 | 0.8 (0.47-1.36) | 0.540 | 1.45 (0.82-2.57) | 0.281 |
| PC 38:2 | 0.4 (0.19-0.83) | 0.057 | **3.76 (1.49-9.49)** | **0.043** |
| PC 38:3 | **3.1 (1.36-7.08)** | **0.039** | **2.85 (1.29-6.26)** | **0.050** |
| PC 38:4 | 0.98 (0.53-1.82) | 0.981 | 1.26 (0.61-2.58) | 0.612 |
| PC 38:5 | 0.73 (0.47-1.13) | 0.291 | 1.24 (0.78-1.97) | 0.453 |
| PC 38:6a | 0.72 (0.45-1.15) | 0.298 | 1.19 (0.78-1.82) | 0.496 |
| PC 38:6b | 0.72 (0.45-1.15) | 0.298 | 1.19 (0.78-1.82) | 0.496 |
| PC 38:7 | 0.46 (0.25-0.84) | 0.051 | 1.52 (0.86-2.68) | 0.226 |
| PC 39:5 | 0.82 (0.5-1.36) | 0.570 | 1.48 (0.84-2.6) | 0.265 |
| PC 39:6 | 0.75 (0.39-1.43) | 0.515 | 1.33 (0.75-2.37) | 0.423 |
| PC 39:7 | 0.72 (0.49-1.07) | 0.214 | 1.22 (0.88-1.7) | 0.317 |
| PC 40:4 | 1.25 (0.65-2.42) | 0.623 | **3.8 (1.64-8.8)** | **0.040** |
| PC 40:5 | 1 (0.64-1.56) | 0.997 | 1.43 (0.96-2.13) | 0.146 |
| PC 40:6 | 1.07 (0.64-1.78) | 0.859 | 1.27 (0.8-2) | 0.397 |
| PC 40:7 | 0.57 (0.35-0.93) | 0.079 | 1.53 (0.89-2.64) | 0.204 |
| PC 40:8 | **0.42 (0.22-0.78)** | **0.037** | **3.52 (1.48-8.38)** | **0.043** |
| phosphatidylcholine | **0.43 (0.23-0.8)** | **0.034** | 2.3 (1.14-4.63) | 0.061 |
| PC O-30:0 | 0.66 (0.34-1.27) | 0.333 | 0.79 (0.36-1.71) | 0.619 |
| PC O-32:0 | 0.6 (0.32-1.14) | 0.242 | 1.37 (0.7-2.68) | 0.453 |
| PC O-32:1 | **0.35 (0.16-0.74)** | **0.038** | 0.9 (0.47-1.71) | 0.782 |
| PC O-32:2 | 0.86 (0.59-1.26) | 0.569 | 1.03 (0.79-1.36) | 0.838 |
| PC O-34:0 | 0.81 (0.49-1.31) | 0.515 | 1.69 (0.95-3.02) | 0.146 |
| PC O-34:1 | 0.55 (0.29-1.06) | 0.173 | 0.99 (0.49-1.97) | 0.973 |
| PC O-34:2 | 0.73 (0.34-1.6) | 0.568 | 0.86 (0.48-1.51) | 0.663 |
| PC O-34:3 | 0.9 (0.54-1.5) | 0.772 | 0.99 (0.68-1.44) | 0.962 |
| PC O-34:4 | **0.27 (0.11-0.64)** | **0.024** | 1.4 (0.9-2.18) | 0.217 |
| PC O-35:4 | 0.59 (0.3-1.16) | 0.245 | 1.14 (0.61-2.14) | 0.726 |
| PC O-36:0 | 1.13 (0.82-1.55) | 0.569 | 1.55 (0.87-2.75) | 0.219 |
| PC O-36:3 | 0.84 (0.44-1.62) | 0.716 | 1.56 (0.69-3.56) | 0.376 |
| PC O-36:4 | 0.99 (0.58-1.71) | 0.993 | 0.88 (0.4-1.95) | 0.787 |
| PC O-36:5 | 0.67 (0.36-1.25) | 0.334 | 1.21 (0.7-2.1) | 0.571 |
| PC O-38:4 | 0.86 (0.48-1.52) | 0.710 | 0.9 (0.47-1.72) | 0.780 |
| PC O-38:5 | 0.91 (0.51-1.62) | 0.827 | 0.59 (0.24-1.47) | 0.350 |
| PC O-40:5 | 0.43 (0.2-0.9) | 0.079 | 1.35 (0.58-3.11) | 0.568 |
| PC O-40:6 | 0.64 (0.34-1.23) | 0.311 | 1.05 (0.48-2.29) | 0.915 |
| PC O-40:7 | 0.76 (0.36-1.57) | 0.570 | 0.82 (0.36-1.87) | 0.692 |
| alkylphosphatidylcholine | 0.73 (0.39-1.36) | 0.424 | 0.94 (0.45-1.96) | 0.902 |
| PC P-32:0 | **0.17 (0.06-0.47)** | **0.010** | 1.24 (0.73-2.12) | 0.515 |
| PC P-32:1 | **0.16 (0.07-0.4)** | **0.004** | 1.01 (0.64-1.59) | 0.967 |
| PC P-34:1 | **0.22 (0.09-0.5)** | **0.008** | 1.18 (0.64-2.18) | 0.663 |
| PC P-34:2 | **0.08 (0.02-0.31)** | **0.007** | 1.78 (0.85-3.71) | 0.207 |
| PC P-34:3 | 0.63 (0.33-1.22) | 0.298 | 3.15 (1.19-8.36) | 0.077 |
| PC P-36:2 | **0.21 (0.08-0.54)** | **0.013** | 1.18 (0.63-2.22) | 0.666 |
| PC P-36:4 | **0.28 (0.11-0.7)** | **0.037** | 1.23 (0.62-2.43) | 0.632 |
| PC P-36:5 | 0.52 (0.28-0.96) | 0.103 | 1.29 (0.81-2.04) | 0.372 |
| PC P-38:5 | 0.31 (0.12-0.8) | 0.060 | 1.11 (0.48-2.56) | 0.837 |
| PC P-40:5 | 0.46 (0.22-0.93) | 0.092 | 1.03 (0.5-2.16) | 0.942 |
| PC P-40:6 | **0.28 (0.11-0.69)** | **0.034** | 1.24 (0.57-2.72) | 0.657 |
| alkenylphosphatidylcholine (plasmalogen) | **0.12 (0.04-0.33)** | **0.002** | 1.46 (0.65-3.27) | 0.431 |
| LPC 14:0 | 2.19 (0.82-5.87) | 0.242 | 2.28 (0.97-5.32) | 0.127 |
| LPC 15:0 | **5.01 (1.98-12.67)** | **0.010** | 1.83 (0.88-3.8) | 0.181 |
| LPC 16:0 | 2.52 (1.07-5.95) | 0.098 | 3 (1.2-7.51) | 0.073 |
| LPC 16:1 | 0.75 (0.49-1.14) | 0.313 | 2.86 (1.27-6.46) | 0.054 |
| LPC 17:0 | **3.14 (1.36-7.26)** | **0.040** | 2.28 (1-5.18) | 0.118 |
| LPC 17:1 | 1.22 (0.57-2.63) | 0.714 | **3.52 (1.41-8.76)** | **0.047** |
| LPC 18:0 | 3.23 (1.16-8.96) | 0.079 | 2.14 (1.03-4.44) | 0.105 |
| LPC 18:1 | 0.52 (0.29-0.93) | 0.085 | 2.27 (0.94-5.46) | 0.138 |
| LPC 18:2 | **0.22 (0.09-0.53)** | **0.012** | 1.41 (0.53-3.71) | 0.571 |
| LPC 18:3 | **0.39 (0.19-0.81)** | **0.049** | 1.71 (0.78-3.76) | 0.265 |
| LPC 20:0 | 0.85 (0.52-1.4) | 0.641 | **3.76 (1.49-9.47)** | **0.043** |
| LPC 20:1 | 1 (0.61-1.64) | 0.997 | 3.47 (1.25-9.61) | 0.066 |
| LPC 20:2 | 0.78 (0.46-1.31) | 0.480 | **2.66 (1.28-5.5)** | **0.049** |
| LPC 20:3 | 0.83 (0.45-1.55) | 0.682 | 2.96 (1.27-6.88) | 0.055 |
| LPC 20:4 | 0.63 (0.32-1.26) | 0.324 | 1.33 (0.6-2.95) | 0.563 |
| LPC 20:5 | 0.84 (0.55-1.28) | 0.556 | 1.09 (0.73-1.62) | 0.721 |
| LPC 22:0 | **0.29 (0.13-0.65)** | **0.021** | 1.69 (0.92-3.13) | 0.166 |
| LPC 22:1 | **0.53 (0.33-0.85)** | **0.041** | 1.5 (0.82-2.73) | 0.275 |
| LPC 22:5 | 0.53 (0.3-0.93) | 0.082 | 1.62 (0.83-3.16) | 0.240 |
| LPC 22:6 | 0.63 (0.38-1.07) | 0.190 | 1.33 (0.81-2.17) | 0.350 |
| LPC 24:0 | **0.17 (0.06-0.47)** | **0.010** | 1.89 (0.94-3.8) | 0.143 |
| LPC 26:0 | **0.36 (0.18-0.72)** | **0.028** | 0.88 (0.48-1.62) | 0.726 |
| lysophosphatidylcholine | 1.23 (0.6-2.51) | 0.662 | 2.74 (1.11-6.77) | 0.071 |
| LPC O-16:0 | **3.05 (1.35-6.86)** | **0.039** | 1.73 (0.82-3.64) | 0.229 |
| LPC O-18:0 | 2.11 (1.04-4.26) | 0.105 | 2.2 (1.01-4.82) | 0.117 |
| LPC O-18:1 | 3.13 (1.19-8.28) | 0.073 | 1.61 (0.73-3.55) | 0.333 |
| LPC O-20:0 | 1.3 (0.8-2.12) | 0.420 | 1.68 (0.87-3.25) | 0.204 |
| LPC O-22:0 | 0.8 (0.45-1.42) | 0.570 | 1.29 (0.58-2.87) | 0.608 |
| LPC O-22:1 | 0.64 (0.36-1.14) | 0.245 | 1.19 (0.59-2.4) | 0.690 |
| LPC O-24:0 | 0.58 (0.3-1.12) | 0.214 | 1.51 (0.73-3.14) | 0.355 |
| LPC O-24:1 | **0.41 (0.21-0.81)** | **0.048** | 1.15 (0.55-2.43) | 0.757 |
| LPC O-24:2 | 0.53 (0.29-0.97) | 0.106 | 1.17 (0.6-2.28) | 0.708 |
| lysoalkylphosphatidylcholine (lysoplatelet activating factor) | 2.21 (1.1-4.47) | 0.078 | 1.87 (0.82-4.3) | 0.203 |
| PE 32:0 | **3.32 (1.57-7.01)** | **0.017** | 3.09 (1.23-7.71) | 0.066 |
| PE 32:1 | 0.9 (0.54-1.48) | 0.772 | **3.85 (1.63-9.09)** | **0.040** |
| PE 34:1 | 1.49 (0.78-2.86) | 0.350 | **2.62 (1.31-5.26)** | **0.045** |
| PE 34:2 | 1.68 (0.86-3.3) | 0.245 | **2.79 (1.29-6.01)** | **0.050** |
| PE 34:3 | 1.08 (0.72-1.62) | 0.793 | **3.95 (1.59-9.81)** | **0.040** |
| PE 35:2 | 3.98 (1.29-12.25) | 0.062 | **3.87 (1.65-9.09)** | **0.040** |
| PE 36:0 | 2.13 (1.07-4.25) | 0.092 | 1.18 (0.62-2.26) | 0.682 |
| PE 36:1 | 2.27 (0.79-6.57) | 0.246 | **3.26 (1.48-7.18)** | **0.040** |
| PE 36:2 | 2.18 (0.93-5.14) | 0.173 | **2.91 (1.41-6.02)** | **0.041** |
| PE 36:3 | 1.64 (0.82-3.28) | 0.291 | **2.51 (1.26-4.98)** | **0.049** |
| PE 36:4 | 1.54 (0.88-2.69) | 0.245 | **3.2 (1.46-7.02)** | **0.041** |
| PE 36:5 | 1.14 (0.71-1.81) | 0.710 | 1.72 (1.04-2.84) | 0.096 |
| PE 38:3 | 2.02 (1.1-3.71) | 0.079 | **4.36 (1.84-10.37)** | **0.040** |
| PE 38:4 | 1.63 (0.99-2.68) | 0.138 | 2.27 (1.13-4.58) | 0.077 |
| PE 38:5 | 1.62 (0.92-2.84) | 0.200 | **3.3 (1.54-7.08)** | **0.040** |
| PE 38:6 | 1.59 (0.94-2.69) | 0.183 | 1.87 (0.97-3.58) | 0.131 |
| PE 40:5 | 2.49 (1.16-5.32) | 0.068 | **3.79 (1.59-9.04)** | **0.040** |
| PE 40:6 | 2.2 (1.19-4.05) | 0.051 | 2.07 (1.07-4) | 0.091 |
| PE 40:7 | 1.25 (0.82-1.93) | 0.436 | 1.98 (1.05-3.73) | 0.094 |
| phosphatidylethanolamine | 2.12 (1.04-4.32) | 0.098 | **4.42 (1.79-10.92)** | **0.020** |
| PE O-34:1 | 0.73 (0.42-1.27) | 0.394 | 1.54 (0.79-3.01) | 0.288 |
| PE O-34:2 | 0.77 (0.41-1.46) | 0.564 | 1.38 (0.73-2.62) | 0.408 |
| PE O-36:2 | 0.63 (0.33-1.21) | 0.298 | 1.93 (0.96-3.87) | 0.133 |
| PE O-36:3 | 0.69 (0.36-1.32) | 0.388 | 1.9 (0.99-3.66) | 0.123 |
| PE O-36:4 | 0.87 (0.45-1.7) | 0.778 | 1.44 (0.62-3.34) | 0.493 |
| PE O-36:5 | 0.66 (0.36-1.2) | 0.300 | 1.65 (0.95-2.84) | 0.144 |
| PE O-36:6 | 0.69 (0.39-1.23) | 0.334 | 2.39 (1.11-5.13) | 0.082 |
| PE O-38:4 | 0.9 (0.55-1.48) | 0.775 | 1.79 (0.95-3.37) | 0.140 |
| PE O-38:5 | 0.65 (0.34-1.26) | 0.328 | 1.62 (0.75-3.5) | 0.305 |
| PE O-40:5 | 0.41 (0.18-0.9) | 0.084 | 1.97 (0.8-4.85) | 0.220 |
| PE O-40:6 | 0.52 (0.27-1) | 0.131 | 2.26 (1.08-4.72) | 0.091 |
| PE O-40:7 | **0.37 (0.18-0.78)** | **0.041** | 1.86 (0.87-3.95) | 0.184 |
| alkylphosphatidylethanolamine | 0.65 (0.36-1.18) | 0.323 | 2.24 (1.05-4.78) | 0.081 |
| PE P-34:1 | 0.78 (0.48-1.29) | 0.466 | 1.64 (1.04-2.58) | 0.094 |
| PE P-34:2 | 1.26 (0.7-2.25) | 0.568 | 1.39 (0.89-2.19) | 0.230 |
| PE P-36:1 | 0.74 (0.42-1.3) | 0.435 | 3.38 (1.31-8.76) | 0.056 |
| PE P-36:2 | 0.66 (0.33-1.33) | 0.375 | 2.12 (1.02-4.42) | 0.112 |
| PE P-36:4 | 1.3 (0.86-1.97) | 0.340 | 1.58 (0.96-2.57) | 0.140 |
| PE P-38:4 | 1.14 (0.75-1.72) | 0.653 | 1.93 (0.97-3.84) | 0.132 |
| PE P-38:5 | 0.63 (0.32-1.27) | 0.328 | 2.48 (1.11-5.58) | 0.086 |
| PE P-38:6 | 0.69 (0.39-1.21) | 0.323 | 1.51 (0.79-2.9) | 0.304 |
| PE P-40:5 | 0.51 (0.26-0.99) | 0.121 | 2.48 (1.12-5.5) | 0.082 |
| PE P-40:6 | 0.62 (0.36-1.08) | 0.198 | 2.11 (1.15-3.87) | 0.066 |
| phosphatidylethanolamine (plasmalogen) | 0.88 (0.53-1.45) | 0.662 | 2.03 (1.1-3.77) | 0.067 |
| LPE 16:0 | 1.16 (0.62-2.18) | 0.754 | **3.15 (1.46-6.8)** | **0.040** |
| LPE 18:0 | 2.63 (1.12-6.17) | 0.082 | **4.13 (1.66-10.25)** | **0.040** |
| LPE 18:1 | 0.68 (0.44-1.06) | 0.192 | 2.06 (0.91-4.67) | 0.153 |
| LPE 18:2 | 0.54 (0.33-0.88) | 0.055 | 1.84 (0.92-3.68) | 0.157 |
| LPE 20:4 | 0.56 (0.3-1.06) | 0.178 | 1.78 (0.95-3.31) | 0.141 |
| LPE 22:6 | **0.48 (0.28-0.84)** | **0.044** | 1.53 (0.88-2.66) | 0.209 |
| lysophosphatidylethanolamine | 0.74 (0.44-1.24) | 0.358 | **3.07 (1.33-7.06)** | **0.036** |
| PI 32:0 | 1.73 (0.82-3.66) | 0.280 | 2.88 (1.28-6.48) | 0.052 |
| PI 32:1 | 1.06 (0.56-1.98) | 0.898 | **4.56 (1.71-12.15)** | **0.040** |
| PI 34:0 | 1.44 (0.84-2.47) | 0.313 | 2.41 (1.08-5.34) | 0.091 |
| PI 34:1 | 1.18 (0.58-2.38) | 0.754 | **3.32 (1.4-7.84)** | **0.045** |
| PI 36:1 | 0.81 (0.45-1.46) | 0.604 | 1.91 (0.93-3.93) | 0.148 |
| PI 36:2 | 0.53 (0.3-0.95) | 0.092 | **2.78 (1.36-5.69)** | **0.043** |
| PI 36:3 | 0.7 (0.41-1.21) | 0.328 | **4.6 (1.91-11.11)** | **0.040** |
| PI 36:4 | 0.92 (0.59-1.45) | 0.809 | 2.62 (1.21-5.67) | 0.063 |
| PI 38:2 | 0.81 (0.51-1.28) | 0.496 | 1.43 (0.84-2.45) | 0.275 |
| PI 38:3 | 0.65 (0.32-1.32) | 0.363 | **3.37 (1.37-8.25)** | **0.049** |
| PI 38:4 | 0.67 (0.4-1.12) | 0.245 | 2.09 (1.06-4.14) | 0.094 |
| PI 38:5 | 0.91 (0.59-1.42) | 0.778 | **3.13 (1.38-7.09)** | **0.045** |
| PI 38:6 | 1.23 (0.66-2.27) | 0.637 | 1.77 (1.04-2.99) | 0.094 |
| PI 40:4 | 1 (0.57-1.77) | 0.997 | 2.55 (1.23-5.26) | 0.054 |
| PI 40:5 | 1.01 (0.6-1.71) | 0.988 | 1.88 (1.05-3.34) | 0.094 |
| PI 40:6 | 0.94 (0.6-1.48) | 0.854 | 1.45 (0.96-2.17) | 0.146 |
| phosphatidylinositol | 0.71 (0.42-1.21) | 0.347 | **3.03 (1.53-6.03)** | **0.020** |
| LPI 18:0 | 0.99 (0.67-1.47) | 0.988 | 1.52 (0.95-2.43) | 0.152 |
| LPI 18:1 | 1.09 (0.68-1.74) | 0.796 | 1.69 (1-2.86) | 0.119 |
| LPI 18:2 | 0.88 (0.47-1.63) | 0.775 | 1.84 (0.99-3.45) | 0.125 |
| LPI 20:4 | 1.11 (0.55-2.21) | 0.838 | 1.66 (0.8-3.42) | 0.256 |
| lysophosphatidylinositol | 1.02 (0.63-1.67) | 0.926 | 1.69 (1-2.86) | 0.087 |
| PS 36:1 | 1.26 (0.88-1.81) | 0.332 | 1.56 (0.98-2.49) | 0.132 |
| PS 38:3 | 1.22 (0.9-1.64) | 0.328 | 1.49 (0.93-2.37) | 0.171 |
| PS 38:4 | 1.18 (0.92-1.53) | 0.328 | 1.41 (0.92-2.16) | 0.191 |
| PS 38:5 | 1.25 (0.9-1.72) | 0.313 | 1.32 (0.93-1.89) | 0.204 |
| phosphatidylserine | 1.23 (0.9-1.67) | 0.347 | 1.44 (0.94-2.21) | 0.144 |
| PG 34:1 | 0.98 (0.62-1.54) | 0.952 | **2.57 (1.26-5.23)** | **0.050** |
| PG 36:2 | 1.48 (0.73-3) | 0.403 | 1.84 (0.88-3.84) | 0.184 |
| phosphatidylglycerol | 1.15 (0.61-2.17) | 0.690 | **2.73 (1.25-5.96)** | **0.043** |
| CE 14:0 | 2.53 (1.13-5.67) | 0.079 | **3.85 (1.51-9.82)** | **0.043** |
| CE 15:0 | 1.4 (0.79-2.46) | 0.375 | 1.82 (0.9-3.66) | 0.168 |
| CE 16:0 | 1.44 (0.71-2.92) | 0.445 | 2.02 (0.99-4.11) | 0.121 |
| CE 16:1 | 1.23 (0.65-2.33) | 0.641 | **4.08 (1.63-10.16)** | **0.040** |
| CE 16:2 | 1.33 (0.76-2.3) | 0.447 | **3.19 (1.47-6.89)** | **0.040** |
| CE 17:0 | 1.09 (0.62-1.92) | 0.827 | 1.55 (0.81-2.98) | 0.274 |
| CE 17:1 | 1.05 (0.58-1.89) | 0.918 | 2.22 (1.15-4.31) | 0.070 |
| CE 18:0 | 0.94 (0.54-1.65) | 0.890 | 1.77 (0.87-3.62) | 0.194 |
| CE 18:1 | 0.75 (0.43-1.31) | 0.439 | 2.07 (0.98-4.39) | 0.127 |
| CE 18:2 | 1.88 (1.03-3.45) | 0.110 | **3.7 (1.39-9.83)** | **0.049** |
| CE 18:3 | 0.96 (0.63-1.45) | 0.891 | **4.44 (1.66-11.89)** | **0.040** |
| CE 20:1 | 0.65 (0.38-1.13) | 0.245 | 1.26 (0.76-2.09) | 0.468 |
| CE 20:2 | **4.92 (1.99-12.15)** | **0.010** | **4.31 (1.78-10.44)** | **0.040** |
| CE 20:3 | 1.31 (0.67-2.56) | 0.568 | 2.15 (1.12-4.13) | 0.077 |
| CE 20:4 | 1.07 (0.58-1.95) | 0.890 | 2.79 (1.22-6.34) | 0.064 |
| CE 20:5 | 1.07 (0.82-1.39) | 0.739 | 1.16 (0.89-1.51) | 0.372 |
| CE 22:0 | 0.89 (0.41-1.93) | 0.834 | 2.29 (0.78-6.69) | 0.211 |
| CE 22:1 | 0.73 (0.39-1.36) | 0.450 | 1.52 (0.9-2.57) | 0.194 |
| CE 22:4 | 2.91 (1.24-6.82) | 0.057 | 2.25 (0.94-5.34) | 0.137 |
| CE 22:5 | 1.09 (0.7-1.72) | 0.785 | 1.59 (0.96-2.65) | 0.144 |
| CE 22:6 | 1.11 (0.75-1.64) | 0.710 | 1.48 (0.95-2.3) | 0.153 |
| CE 24:0 | 0.93 (0.58-1.48) | 0.827 | 1.73 (0.87-3.45) | 0.198 |
| CE 24:6 | 1.41 (0.85-2.33) | 0.314 | **2.22 (1.26-3.92)** | **0.045** |
| cholesteryl ester | 1.35 (0.7-2.62) | 0.453 | **3.62 (1.51-8.69)** | **0.022** |
| free cholesterol (ST 27:1/OH) | 0.7 (0.39-1.24) | 0.340 | 1 (0.99-1.01) | 0.879 |
| DG 14:0_16:0 | **7.36 (2.08-26.1)** | **0.019** | 1.98 (1.17-3.34) | 0.052 |
| DG 14:0_18:1 | **5.24 (1.72-15.99)** | **0.026** | **2.67 (1.29-5.53)** | **0.049** |
| DG 14:0_18:2 | **4.99 (1.98-12.58)** | **0.010** | 2.12 (1.05-4.26) | 0.095 |
| DG 16:0_16:0 | **16.82 (4.24-66.68)** | **0.004** | 2.53 (1.23-5.21) | 0.054 |
| DG 16:0_18:0 | **24.44 (5.42-110.27)** | **0.004** | 2.22 (1.15-4.29) | 0.068 |
| DG 16:0_18:1 | **18.52 (5.25-65.37)** | **0.004** | **4.36 (1.64-11.65)** | **0.040** |
| DG 16:0_18:2 | **10.17 (3.38-30.58)** | **0.004** | 2.4 (1.15-5.02) | 0.074 |
| DG 16:0_20:4 | **7.93 (2.96-21.2)** | **0.004** | 2.09 (1.14-3.81) | 0.066 |
| DG 16:0_22:5 | **3.82 (1.77-8.24)** | **0.010** | 1.8 (1.05-3.09) | 0.094 |
| DG 16:1_18:1 | 1.82 (0.91-3.66) | 0.198 | **4.1 (1.54-10.96)** | **0.043** |
| DG 18:0_18:1 | **8.23 (2.27-29.85)** | **0.014** | **4.65 (1.68-12.88)** | **0.040** |
| DG 18:0_18:2 | **8.13 (2.66-24.86)** | **0.007** | 2.03 (1.06-3.89) | 0.093 |
| DG 18:0_20:4 | **2.36 (1.25-4.46)** | **0.041** | 1.99 (1.05-3.77) | 0.094 |
| DG 18:1_18:1 | 2.71 (1.19-6.21) | 0.067 | **3.82 (1.61-9.07)** | **0.040** |
| DG 18:1_18:2 | 2.24 (1.14-4.38) | 0.068 | 2.46 (1.15-5.26) | 0.075 |
| DG 18:1_18:3 | 2.11 (1.05-4.23) | 0.099 | 1.84 (0.99-3.43) | 0.121 |
| DG 18:1_20:3 | 1.48 (0.87-2.52) | 0.278 | 1.78 (1.06-2.98) | 0.089 |
| DG 18:1_20:4 | **2.66 (1.31-5.38)** | **0.038** | 1.59 (1-2.53) | 0.118 |
| DG 18:2_18:2 | 1.77 (0.98-3.19) | 0.146 | 1.32 (0.81-2.17) | 0.355 |
| diacylglycerol | **5.42 (2.06-14.22)** | **0.006** | **3.72 (1.55-8.9)** | **0.022** |
| TG 14:0_16:0_18:1 | **3.65 (1.37-9.76)** | **0.046** | **2.75 (1.31-5.8)** | **0.049** |
| TG 14:0_16:0_18:2 | 2.44 (1.01-5.85) | 0.121 | **4.03 (1.6-10.16)** | **0.040** |
| TG 14:0_16:1_18:1 | 2.1 (0.82-5.33) | 0.242 | **5.39 (1.8-16.13)** | **0.040** |
| TG 14:0_16:1_18:2 | 2.79 (1.22-6.37) | 0.059 | 2.86 (1.2-6.81) | 0.070 |
| TG 14:0_17:0_18:1 | **3.82 (1.73-8.44)** | **0.013** | **2.71 (1.35-5.46)** | **0.043** |
| TG 14:0_18:0_18:1 | 4.29 (0.68-26.93) | 0.242 | 1.74 (1.01-2.98) | 0.111 |
| TG 14:0_18:2_18:2 | 2.06 (1.1-3.84) | 0.079 | 2.24 (1.02-4.94) | 0.111 |
| TG 14:1_16:0_18:1 | 1.67 (0.74-3.76) | 0.340 | **5.51 (1.85-16.42)** | **0.040** |
| TG 14:1_16:1_18:0 | 1.08 (0.54-2.16) | 0.886 | **5.47 (1.89-15.86)** | **0.040** |
| TG 14:1_18:0_18:2 | 1.19 (0.62-2.32) | 0.711 | **8.44 (2.63-27.1)** | **0.040** |
| TG 14:1_18:1_18:1 | 1.43 (0.75-2.75) | 0.407 | **7.89 (2.29-27.23)** | **0.040** |
| TG 15:0_16:0_18:1 | **3.89 (1.63-9.26)** | **0.019** | **2.77 (1.35-5.69)** | **0.045** |
| TG 15:0_18:1_18:1 | **2.74 (1.28-5.85)** | **0.044** | **5.65 (1.92-16.69)** | **0.040** |
| TG 16:0_16:0_16:0 | **3.12 (1.45-6.69)** | **0.026** | 1.9 (1.03-3.51) | 0.101 |
| TG 16:0_16:0_18:0 | **5.72 (2.2-14.88)** | **0.008** | 1.7 (1-2.88) | 0.119 |
| TG 16:0_16:0_18:1 | **3.7 (1.54-8.85)** | **0.025** | **3.1 (1.35-7.11)** | **0.049** |
| TG 16:0_16:0_18:2 | **4.32 (1.87-9.97)** | **0.010** | 2.52 (1.24-5.1) | 0.052 |
| TG 16:0_16:1_17:0 | **3.99 (1.75-9.09)** | **0.013** | **3.25 (1.42-7.39)** | **0.043** |
| TG 16:0_16:1_18:1 | 1.66 (0.78-3.52) | 0.313 | **9.56 (2.44-37.4)** | **0.040** |
| TG 16:0_17:0_18:0 | **8.47 (2.94-24.43)** | **0.004** | 1.66 (0.97-2.83) | 0.133 |
| TG 16:0_17:0_18:1 | **7.06 (2.21-22.58)** | **0.013** | **2.89 (1.41-5.89)** | **0.041** |
| TG 16:0_17:0_18:2 | **3.11 (1.41-6.88)** | **0.032** | **9.53 (2.48-36.64)** | **0.040** |
| TG 16:0_18:0_18:1 | **4.93 (1.54-15.82)** | **0.039** | 1.97 (1.09-3.54) | 0.081 |
| TG 16:0_18:1_18:1 | 2.07 (0.99-4.35) | 0.138 | **7.31 (2-26.7)** | **0.040** |
| TG 16:0_18:1_18:2 | 1.91 (0.99-3.68) | 0.138 | **3.61 (1.51-8.62)** | **0.041** |
| TG 16:0_18:2_18:2 | 1.95 (1.05-3.61) | 0.099 | 2.24 (1.12-4.47) | 0.078 |
| TG 16:1_16:1_16:1 | 0.84 (0.46-1.55) | 0.699 | **6.59 (2.11-20.58)** | **0.040** |
| TG 16:1_16:1_18:0 | 3.88 (0.69-21.86) | 0.245 | 2.12 (1.15-3.91) | 0.066 |
| TG 16:1_16:1_18:1 | 1.06 (0.59-1.88) | 0.891 | **8.08 (2.43-26.94)** | **0.040** |
| TG 16:1_17:0_18:1 | **2.74 (1.3-5.78)** | **0.041** | **7.67 (2.23-26.41)** | **0.040** |
| TG 16:1_18:1_18:1 | 0.89 (0.52-1.52) | 0.772 | **7.65 (2.29-25.54)** | **0.040** |
| TG 16:1_18:1_18:2 | 1.43 (0.75-2.72) | 0.407 | **5.18 (1.81-14.78)** | **0.040** |
| TG 17:0_18:1_18:1 | 2.07 (1.1-3.88) | 0.079 | **6.9 (2.15-22.17)** | **0.040** |
| TG 18:0_18:0_18:0 | **7.99 (2.89-22.09)** | **0.004** | 1.3 (0.77-2.22) | 0.423 |
| TG 18:0_18:0_18:1 | 8.66 (1.47-51.18) | 0.065 | 1.43 (0.95-2.13) | 0.153 |
| TG 18:0_18:1_18:1 | 3.31 (0.82-13.4) | 0.200 | **2.92 (1.32-6.48)** | **0.049** |
| TG 18:0_18:2_18:2 | 2.73 (1.25-5.94) | 0.051 | 1.7 (0.9-3.19) | 0.175 |
| TG 18:1_18:1_18:1 | 1.25 (0.7-2.24) | 0.575 | **2.8 (1.32-5.95)** | **0.049** |
| TG 18:1_18:1_18:2 | 1.45 (0.79-2.67) | 0.354 | 2.43 (1.1-5.34) | 0.086 |
| TG 18:1_18:1_20:4 | 1.59 (0.94-2.71) | 0.191 | 2.14 (1.05-4.35) | 0.095 |
| TG 18:1_18:1_22:6 | 1.6 (0.91-2.79) | 0.214 | 2.11 (1.05-4.25) | 0.098 |
| TG 18:1_18:2_18:2 | 1.36 (0.84-2.19) | 0.333 | 1.8 (0.93-3.49) | 0.152 |
| TG 18:2_18:2_18:2 | 1.51 (0.92-2.49) | 0.217 | 1.24 (0.78-1.96) | 0.453 |
| TG 18:2_18:2_20:4 | 1.5 (0.96-2.36) | 0.178 | 1.34 (0.81-2.23) | 0.341 |
| triaclyglycerol | 2.48 (1.15-5.38) | 0.070 | **5.94 (1.89-18.66)** | **0.020** |
| ***^1^*** *Cer, ceramide; CE, cholesteryl ester; DG, diacylglycerol; GM3, G_M3_ ganglioside; HexCer, monohexosylceramide; Hex2Cer, dihexosylceramide; Hex3Cer, trihexosylceramide; LPC, lysophosphatidylcholine; LPC O, lysoalkylphosphatidylcholine (lysoplatelet activating factor); LPE, lysophosphatidylethanolamine; LPI, lysophosphatidylinositol; PC, phosphatidylcholine; PC O, alkylphosphatidylcholine; PC P, alkenylphosphatidylcholine (plasmalogen); PE, phosphatidylethanolamine; PE O, alkylphosphatidylethanolamine; PE P, alkenylphosphatidylethanolamine (plasmalogen); PG, phosphatidylglycerol; PI, phosphatidylinositol; PS, phosphatidylserine; SM, sphingomyelin; TG, triacylglycerol. Full names represent the sum of the individual species within a class or subclass.*  ***^2^*** *mean (95% confidence interval) adjusted for current statin treatment and corresponding to on an inter-quartile range increase in the predictor lipid species measurement*  ***^3^*** *false discovery rate-corrected p-values using the Benjamini-Hochberg method* | | | | |
